# Supplementary material for: Reliability and clinical correlations of semi-quantitative lung ultrasound on BLUE points in COVID-19 mechanically ventilated patients: The ‘BLUE-LUSS’—A feasibility clinical study
Source: PLoS One. 2022 Oct 14;17(10):e0276213. doi: 10.1371/journal.pone.0276213 (PMC9565374; doi:10.1371/journal.pone.0276213)
Supplement: S1 Table — (DOCX) [file pone.0276213.s001.docx]

**S1 Table. Correlations of LUSS scores and PCT, APACHE II and CURB 65 Pneumonia Score**

| **LUSS** | **variable** | **Pearson’s *r*** | **p value** |
| --- | --- | --- | --- |
| **cLUSS longitudinal** | PCT | -0.18 | 0.4262 |
|  | APACHE II | -0.16 | 0.5062 |
|  | CURB 65 | -0.11 | 0.6357 |
| **cLUSS parallel** | PCT | -0.20 | 0.3990 |
|  | APACHE II | -0.16 | 0.4940 |
|  | CURB 65 | -0.12 | 0.6063 |
| **qLUSS longitudinal** | PCT | -0.20 | 0.4056 |
|  | APACHE II | -0.24 | 0.3031 |
|  | CURB 65 | -0.15 | 0.5182 |
| **qLUSS parallel** | PCT | -0.03 | 0.8933 |
|  | APACHE II | -0.21 | 0.3817 |
|  | CURB 65 | -0.18 | 0.4553 |
